# Blowhole Colostomy for* Clostridium difficile*-Associated Toxic Megacolon

**DOI:** 10.1155/2016/5909248

**Published:** 2016-12-21

**Authors:** Jeroen Kerstens, Ian Diebels, Charles de Gheldere, Patrick Vanclooster

**Affiliations:** ^1^Faculty of Medicine and Health Sciences, University of Antwerp, Edegem, Belgium; ^2^Department of General Surgery, Heilig-Hartziekenhuis, Lier, Belgium

## Abstract

We present the case of a 58-year-old man who underwent urgent blowhole colostomy for toxic megacolon (TM) secondary to* Clostridium difficile* infection (CDI). This infection occurred under antibiotic coverage with amoxicillin-clavulanic acid, four days after laparoscopic sigmoidectomy in our hospital. Although prospective clinical research regarding the surgical management of TM is lacking, decompressive procedures like blowhole colostomy are reported to carry a high risk of postoperative morbidity and mortality and are widely regarded as obsolete. Subtotal or total colectomy with end ileostomy is currently considered the procedure of choice. After presenting our case, we discuss the literature available on the subject to argue that the scarce evidence on the optimal surgical treatment for TM is primarily based on TM associated with inflammatory bowel diseases (IBD) and that there might be a rationale for considering minimally invasive procedures like blowhole colostomy for CDI-associated TM.

## 1. Introduction

Toxic megacolon (TM) is a rare but potentially life-threatening complication of acute severe colitis. Its main characteristics are radiographic evidence of total or segmental colonic distension of >6 cm without mechanical obstruction and the additional presence of systemic toxicity. The most commonly used clinical criteria for the diagnosis of TM were proposed by Jalan et al. (1969) [1]. Three out of four of the following are mandatory for diagnosis: fever (>38.6°C), tachycardia (>120 bpm), leukocytosis (>10.5 · 10^3^/*μ*L), and anaemia (haemoglobin or haematocrit level <60% of normal value). In addition, one of the following should be met: dehydration, altered level of consciousness, electrolyte imbalances, and hypotension. First recognized as a clinical entity by Marshak and Lester (1950) in patients with ulcerative colitis (UC) [2], TM was classically thought to be a complication solely of inflammatory bowel disease (IBD). However, over the course of the last decades, it has become clear that almost any inflammatory condition of the colon can be complicated by toxic dilation [3]. This includes infectious colitis, of which* Clostridium difficile* infection (CDI) is the best known and most prevalent. Moreover, the epidemiology of TM lately has shifted from inflammatory towards infectious causes. Because of earlier recognition and intensive management of UC, both incidence and mortality of UC-associated TM have declined in recent years. Meanwhile, the incidence, severity, and mortality rates of CDI have rapidly increased, with more patients suffering from a community-acquired CDI and more patients requiring surgical intervention [4].

## 2. Case Report

A 58-year-old man was admitted to our hospital for laparoscopic sigmoidectomy and gastrointestinal continuity repair. Three months earlier, he underwent laparoscopic drainage of a parasigmoidal abcess and lateral colostomy for colonic obstruction caused by Hinchey II sigmoid diverticulitis. During this initial admission, he had been treated with amoxicillin-clavulanic acid for two days, after which he was switched to piperacillin-tazobactam due to preoperative clinical deterioration and increasing C-reactive protein (384.4 mg/L; normal range: 0–10). After drainage and colostomy, piperacillin-tazobactam was continued until microbiological examination confirmed the presence of amoxicillin-clavulanic acid-resistant, ciprofloxacin-sensitive* E. coli*. The patient was then switched to oral ciprofloxacin, which was given for a total of twelve days. He recovered quickly.

**Figure 1:**
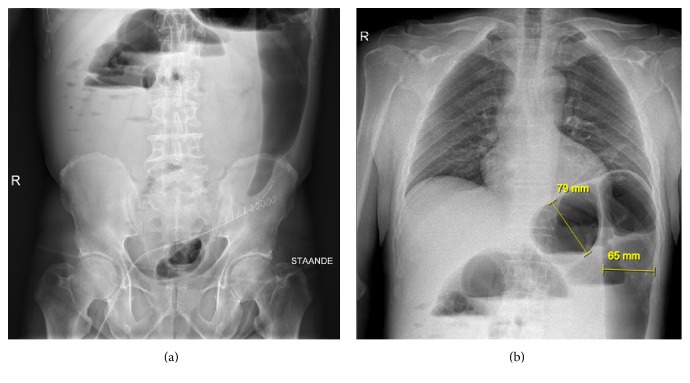
Plain X-ray in standing position showing aerocoly in the epigastric region and the splenic flexure. No evidence of free air under the diaphragm.

**Figure 2:**
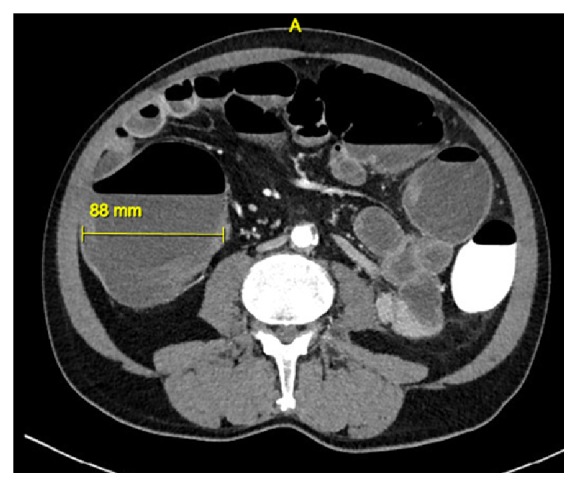
Abdominal CT scan of the abdomen after 300 mL rectal gastrografin and 100 mL intravenous nonionized iodine contrast. Status after partial sigmoidectomy. Gross colonic distension (Ø 88 mm), especially marked at the splenic flexure, without pathological bowel wall thickening. Distension of the terminal ileum, with signs of wall thickening. No free air or fluid, no suture leakage, no abscess, and no signs of ischemia.

Three months later, the patient was thus readmitted for elective laparoscopic sigmoidectomy. The colostomy was excised, the left colon was mobilized, and a mechanical double stapling colorectal anastomosis was performed. The procedure was uncomplicated and the patient recovered quickly, receiving prolonged antibiotic prophylaxis with amoxicillin-clavulanic acid for 48 hours because he was subfebrile after the first 24 hours. However, four days postoperatively, he developed high fever, epigastric pain, nausea, and right-sided acute lower back pain. Clinical examination showed a pale patient with a painful abdomen and normal bowel sounds at auscultation, without signs of peritoneal irritation. Initial blood panel revealed no disturbances other than mild hyponatremia (135 mmol/L; normal range: 137–145) and raised C-reactive protein (337.6 mg/L). Plain X-rays of the abdomen (Figure 1(a)) and thorax (Figure 1(b)) and subsequent abdominal CT scan with intravenous contrast (Figure 2) revealed gross distension of both small and large bowel without signs of perforation.

Within several hours, the patient's condition further deteriorated with rapidly increasing abdominal distension and evolution towards septic shock (temperature: 38.8°C, heart rate: 131 bpm). When his blood pressure started to drop, he was transferred to the intensive care unit. At admission, he was anaemic (haemoglobin: 11.5 g/dL; normal range: 13.1–17.3) and severely acidic (pH 7.30 [normal range: 7.35–7.45]; lactic acid 56.2 mg/dL [normal range: 6.3–18.9]). Colonoscopy was not performed because the risk of perforation was deemed too high. After initial fluid resuscitation, the patient was brought to the operating theater for explorative laparoscopy, which confirmed distension of both small and large bowel without mechanic obstruction, significant edema, ischemia, or serosal tears. A blowhole colostomy was created at the transverse colon via small laparotomy in the right upper quadrant. Screening of peroperatively collected stool samples for* C. difficile* glutamate dehydrogenase antigen and ELISA for* C. difficile* toxins A and B both turned out positive. Faeces cultures were positive for* C. difficile*, thus confirming the diagnosis of* C. difficile* enterocolitis. The patient was placed in quarantine with administration of intravenous metronidazole and piperacillin-tazobactam, the latter of which was stopped when culture results were available. An overview of antibiotics used is given in Table 1.

Two days postoperatively, the patient's condition improved with rapid regression of the septic shock. Further hospitalization was uncomplicated and the patient was transferred to the general surgery ward. Oral feeding was reintroduced and metronidazole administration was switched from intravenous to oral and rectal. After three days of solid stool production, strict quarantine treatment of the patient was no longer necessary. The patient left the hospital in good health on the twelfth postoperative day, continuing his antibiotic treatment for two more days. He returned three months later for gastrointestinal continuity repair under antibiotic prophylaxis (2 grams of cefazolin-metronidazole) and left the hospital fever-free and in good health after three days. He presented without complaints on control visits three days and four weeks later.

## 3. Discussion

Regarding the medical versus surgical management of fulminant colitis and more specifically of TM, prospective studies are largely lacking and the available literature is equivocal [3]. However, there is a trend towards early surgical intervention, especially in case of rapid clinical deterioration and the presence of signs of end-organ failure [5]. Over the years, different surgical approaches have been developed to deal with TM, (sub)total colectomy with end ileostomy being the technique of choice nowadays [4]. A blowhole colostomy procedure for TM was first suggested by Turnbull et al. in 1971 [6]. The authors advocated temporary colonic decompression and diversion by skin-level, cutaneous (blowhole) colostomy or loop ileostomy, with secondary colectomy three to six months later.

Using the Turnbull approach on critically ill patients with UC-associated TM, good results were reported in the 1970s and 1980s [6, 7]. By the end of the 20th century, progress in intensive medical therapy, however, had made Turnbull colostomy a largely obsolete procedure in favor of immediate (sub)total colectomy with end ileostomy [8]. More recently, Ausch et al. published a retrospective study in which seventy patients with TM (46% associated with UC, 31% with CDI, and 23% with other causes) underwent different types of surgeries between 1985 and 2004 [3]. Decompressive procedures (i.e., Turnbull procedure or faecal diversion by transversostomy or caecostomy) were associated with severe postoperative bleeding from the colon in 86% and a total mortality of 71%, compared to 21% postoperative morbidity and mortality in the total colectomy group and only 8% and 6% in the subtotal colectomy group, respectively. Based on these results, the authors concluded that there is no place for decompressive procedures in the surgical treatment of TM [3]. In this context, it is worth mentioning that the report by Ausch et al. does not allow determining the etiology of TM in the patients that received colectomy versus those who received a decompressive procedure.

A more positive account of blowhole colostomy in recent times is given by an American center in a case series of seventeen patients that underwent this procedure with or without ileostomy between 1983 and 2001 [9]. In the absence of necrosis or perforation, this technique gave excellent chances of uncomplicated repair of gastrointestinal continuity. The authors concluded that the blowhole procedure is still indicated for selected patients with TM in high risk situations that specifically display the need for assessment of its place as an alternative to colectomy in* C. difficile* colitis refractory to medical treatment. However, an important limitation of this study is the small number of patients (*n* = 17) that were enrolled in the time frame of 18 years.

Finally, in a retrospective cohort study, Neal et al. reported on diverting loop ileostomy and colonic lavage for the management of fulminant CDI [10]. The authors state that while the infected colon is a major source of systemic inflammation and colectomy may be able to reverse this inflammatory status more rapidly, the invasive nature of colectomy also poses other challenges to critically ill patients. If the colon is not perforated or necrotic, as was the case in our patient, a colon-preserving alternative to colectomy can be considered in patients with fulminant CDI.

Turnbull's initial approach advocated minimal colonic manipulation and immediate stoma creation via paramedian laparotomy in order to prevent further deterioration by disruption of sealed-off perforations. In our case, however, we opted for a preliminary diagnostic laparoscopy to assess the colonic viability and rule out iatrogenic serosal tears or perforation. It was only after confirmation of a viable colon and identification of the optimal stoma site that we felt secure to procede with creating a blowhole colostomy instead of colectomy. In light of the original no-touch approach, diagnostic laparoscopy was performed by an experienced laparoscopic surgeon, minimizing colon manipulation [6, 11].

In summary, we have described a case of CDI-associated TM occurring only four days after laparoscopic sigmoidectomy. A possible contributing factor to the short time frame in which our patient developed this condition is his exposure to a prolonged prophylactic antibiotic regimen of 48 hours. The patient was successfully treated by creating a blowhole colostomy, which provided a venting mechanism for the toxin-related distention. CDI is a toxin-mediated disease that induces a local and systemic inflammatory response on the colonic mucosa. Similar to diverting loop ileostomy and colonic lavage, blowhole colostomy can potentially reverse the pathologic process while preserving the viable colon by interrupting the faecal stream, depriving the luminal flora of nutrition, and removing bacteria and toxins [10].

We are well aware that one case report with a favorable outcome can be the result of simple luck and in itself is insufficient evidence to question the position of (sub)total colectomy with end ileostomy as the gold standard surgical procedure in TM. Indeed, the patient in our case could have benefited equally well from colectomy. Especially in the IBD-associated form in which resection of the chronically inflamed diseased bowel is the only definitive curative option, blowhole colostomy may unnecessarily delay colectomy. However, with this case, we want to add to the ongoing debate concerning the surgical management of CDI-associated TM, given that (a) to the best of our knowledge, there are no prospective studies comparing different surgical approaches in patients with TM in general and CDI-associated TM in specific; (b) the scarce literature concerned with the question of optimal surgical treatment of TM is largely based on patients with IBD-associated TM; (c) decompressive procedures like blowhole colostomy were considered obsolete and therefore rarely performed before the rapid increase in prevalence of CDI and CDI-associated TM became evident; and (d) colectomy in CDI-associated TM only marginally improves survival while being the cause of considerable morbidity with a high number of survivors requiring permanent ileostomy.

In conclusion, there is a pressing need for well-designed, prospective research addressing the question of which method(s) is/are the best for the surgical management of CDI-associated TM. The idea that decompressive procedures like blowhole colostomy are obsolete in this regard may be premature, and minimally invasive procedures should not be overlooked in the search for the optimal surgical treatment for this ever more prevalent condition.

## Figures and Tables

**Table 1:** Antibiotic usage.

Time point	Antibiotic (posology)	Comments
Laparoscopic sigmoidectomy POD0	Amoxicillin-clavulanic acid (1 × 1 gram IV) Amoxicillin-clavulanic acid (1 × 1 gram IV)	Peroperatively
POD1	Amoxicillin-clavulanic acid (4 × 1 gram IV)	
POD2	Amoxicillin-clavulanic acid (2 × 1 gram IV)	
POD3	None	
Blowhole colostomy	Piperacillin-tazobactam (1 × 4 grams IV) Metronidazole (1 × 500 milligrams IV)	Peroperatively
POD1^*∗*^	Piperacillin-tazobactam (4 × 4 grams IV) Metronidazole (3 × 500 milligrams IV)	
POD2^*∗*^	Piperacillin-tazobactam (3 × 4 grams IV) Metronidazole (3 × 500 milligrams IV) Tigecycline (1 × 100 milligrams IV)	
POD3-POD4^*∗*^	Tigecycline (1 × 100 milligrams IV) Metronidazole (3 × 500 milligrams IV)	
POD5–POD7^*∗*^	Metronidazole (3 × 500 milligrams IV)	
POD8–POD14^*∗*^	Metronidazole (3 × 500 milligrams PO)	Discharged on POD12^*∗*^ Stop antibiotics on POD14^*∗*^

IV: intravenous; PO: per os; POD: postoperative day. Postoperative days after the second procedure are marked with an asterisk.

## Abbreviations

CDI: * Clostridium difficile* infection
IBD: Inflammatory bowel disease
TM: Toxic megacolon
UC: Ulcerative colitis.
